# Beyond the Scalpel: A Cross‐Sectional Study of Psychological Well‐Being and Quality of Life in Women With a History of Cosmetic Surgery, Surgical Candidates, and a Comparison Group

**DOI:** 10.1002/hsr2.73245

**Published:** 2026-09-14

**Authors:** Azita Fathnezhad‐Kazemi, Asal Rahbar Zeraati

**Affiliations:** ^1^ Department of Midwifery, TaMS.C. Islamic Azad University Tabriz Iran

**Keywords:** appreciation, body image, cosmetic surgery, fear, psychological, quality of life

## Abstract

**Background and Aims:**

Cosmetic surgery is increasingly performed worldwide and is often sought to address concerns related to body image, which may influence psychological well‐being and quality of life. This study aimed to compare quality of life, psychological well‐being, body appreciation, and body image concerns among women with a history of cosmetic surgery, cosmetic surgery candidates, and a comparison group.

**Methods:**

This cross‐sectional study was conducted among 375 women in Tabriz, Iran. Participants were allocated into three groups: women with a history of cosmetic surgery, women who were candidates for cosmetic surgery, and a comparison group. Data were collected using a demographic questionnaire, the Short Form Health Survey (SF‐12), the General Health Questionnaire (GHQ‐28), the Body Appreciation Scale (BAS), and the Body Image Concern Inventory (BICI). Data were analyzed using one‐way analysis of variance, Tukey's post hoc test, Pearson's correlation, and multiple linear regression.

**Results:**

The mean scores for quality of life, general health, body appreciation, and body image concern were 56.18, 27.58, 50.25, and 41.11, respectively. Significant differences were observed among the three groups in quality of life (*p* < 0.001), general health (*p* < 0.001), body image concern (*p* = 0.004), and body appreciation (*p* = 0.040). Post hoc tests showed that the operated group had more favorable quality‐of‐life and psychological‐health scores and lower body‐image concern than the other two groups; body‐appreciation scores were lowest in the candidate group.

**Conclusion:**

Women with a history of cosmetic surgery reported a more favorable quality of life, body appreciation, body image concerns, and psychological well‐being than cosmetic surgery candidates and women in the comparison group. Routine psychological assessment and careful evaluation of patients' motivations before cosmetic surgery may facilitate informed clinical decision‐making and appropriate patient selection.

**Abbreviations**
BAS: Body Appreciation Scale
BICI: Body Image Concerns Inventory
GHQ‐28: General Health Questionnaire
SF‐12: Short Form Health‐Related Quality‐of‐Life Questionnaire

## 1 Introduction

In recent years, plastic surgery has shifted from primarily addressing congenital and reconstructive issues to increasingly focusing on aesthetic purposes [1, 2]. Annually, tens of thousands of individuals undergo cosmetic surgery aimed at enhancing their physical appearance [3]. In 2010 alone, over 13 million cosmetic plastic surgeries were performed in the United States [2], and the global demand for such procedures continues to rise [1]. The tendency toward cosmetic surgery has significantly increased, particularly in developing countries and among young adults and women of reproductive age [4]. According to available data, the highest rates of cosmetic surgery are observed among women aged 20 to 50 years [1, 5]. Among these procedures, rhinoplasty is the most commonly requested type of plastic surgery [6]. However, other forms of plastic surgery, including breast augmentation, liposuction, blepharoplasty, and minimally invasive techniques (e.g., Botox injections, dermal fillers), are also widely performed (6), with their numbers increasing steadily [7].

Some researchers associate the growing demand for cosmetic surgery with negative body image [8, 9], as many candidates believe that achieving an ideal physical appearance may significantly improve their emotional well‐being and social functioning [10]. Body image is a dynamic construct shaped by cultural, social, and personal factors [2, 11]. Disturbances in body image have been associated with psychological distress, reduced quality of life, and an increased desire for cosmetic surgery [12, 13]. In other words, body image is a dynamic construct [14]. The ideal body image that individuals hold is influenced by cultural norms, social environment, personal traumas, peer attitudes, and other external factors [2].

Studies suggest that there is a correlation between individuals' attitudes toward their bodies and their desire for cosmetic surgery; in general, those with a more positive body image tend to show less inclination toward undergoing surgical interventions [2, 9]. Recent systematic reviews similarly suggest that cosmetic surgery may improve procedure‐specific satisfaction and some psychosocial outcomes; however, evidence regarding long‐term improvements in overall mental health and quality of life remains limited and heterogeneous [15]. As previously mentioned, changes in physical appearance resulting from cosmetic surgery may impact various aspects of an individual's well‐being [9].

Research indicates significant differences in psychological health and body image between individuals seeking cosmetic surgery and those who do not [3, 9]. Researchers have reported that more than half of the candidates for rhinoplasty exhibited at least some abnormal psychological symptoms, with higher prevalence rates compared to the Comparison group [16]. Additionally, studies evaluating the effects of cosmetic surgery have shown variable impacts on patients' quality of life depending on the type of procedure performed [17, 18, 19]. While some studies have demonstrated improvements in quality of life and self‐esteem following surgery, others have failed to confirm such beneficial effects on psychological health or well‐being [20].

Experts emphasize that psychological evaluation, including assessments of mental health, motivation, self‐esteem, and the candidate's perception of their physical appearance, should be conducted before any surgical intervention, following a thorough medical history and physical examination [5]. Given the growing demand for cosmetic surgery, understanding the psychological characteristics and quality of life of women seeking cosmetic surgery is important for appropriate patient selection and clinical decision‐making [21, 22]. Therefore, this study was designed to investigate and compare the health‐related quality of life and psychological well‐being, including physical and mental health, appreciation, and body perception, of individuals seeking cosmetic surgery with that of the Comparison group.

## 2 Methodology

This study was a cross‐sectional investigation conducted from November 1, 2023 to March 31, 2024 on women referring to the beauty clinic and outpatient clinic of Vali‐e‐Asr Hospital in Tabriz, Iran. The study population included three groups: (1) women with a history of previous cosmetic surgery, (2) women seeking cosmetic surgery, and (3) women who had no intention or history of undergoing cosmetic surgery. Women with a history of cosmetic surgery were recruited from the cosmetic surgery clinic during follow‐up visits. Cosmetic surgery candidates were recruited during their preoperative outpatient visits before undergoing the planned procedure. The comparison group consisted of women attending the same hospital outpatient clinic for reasons unrelated to cosmetic surgery who had no history of cosmetic surgery and no intention of undergoing cosmetic surgery.

Inclusion criteria were as follows: Age of 18 years or older; Literacy sufficient to complete self‐reported questionnaires; Absence of chronic medical conditions (e.g., cardiovascular disease, diabetes); No reported history of psychiatric disorders or use of psychotropic medications, based on self‐report; For the surgical group: women who had undergone at least one surgical cosmetic procedure at least 2 months prior, with no functional or trauma‐related indication for the surgery;

For the candidate group: women who had scheduled an appointment for cosmetic surgery; For the comparison group: women attending the same hospital outpatient clinic for reasons unrelated to cosmetic surgery who had no history of cosmetic surgery and no intention of undergoing cosmetic surgery. The comparison group comprised women attending the hospital outpatient clinic who had no history of cosmetic surgery and no intention of undergoing cosmetic surgery. Exclusion criteria included incomplete completion of more than 10% of the questionnaire items and withdrawal of consent to participate in the study. A convenience sampling method was employed, and data collection continued until the calculated sample size was achieved. The previous cosmetic surgery status was determined through participant interviews and verified against available medical records whenever possible. In the comparison group, the absence of previous cosmetic surgery was established through self‐report during participant screening.

### 2.1 Sample Size Calculation

The sample size was determined a priori using a single‐mean precision approach based on the previously reported mean (13.6) and standard deviation (6.4) for general health after cosmetic surgery reported by Zojaji et al. [20]. Assuming a 95% confidence level and a 5% margin of error, the initial required sample size was estimated at 340 participants. To enhance the precision of the study, an additional 10% was added to the sample size, resulting in a final sample size of 375 participants. This calculation was designed to estimate a population mean with a specified precision and was not based on a prespecified effect size or power calculation for the three‐group ANOVA. As a supplementary sensitivity analysis, given the final sample size of 375 participants and the observed group allocation (123, 138, and 114 participants), a one‐way ANOVA with *α* = 0.05 would have 80% power to detect an effect size of approximately Cohen's *f* = 0.16. This sensitivity analysis was conducted solely for transparency and does not represent the original sample‐size justification.

$$n=\frac{{\left(Z_{1}-\frac{\alpha }{2}\right)}^{2}\times s^{2}}{d^{2}}\;$$

### 2.2 Method of Implementation

After obtaining ethical approval and necessary permissions from the authorities at Tabriz University of Medical Sciences and Vali‐e‐Asr Hospital, the researcher visited cosmetic clinics for participant recruitment. Following screening based on inclusion criteria, the objectives of the study were explained to eligible women, and informed consent was obtained. Subsequently, self‐report questionnaires were administered to the participants. The researcher provided guidance as needed in case of any difficulties or questions during questionnaire completion. After obtaining informed consent, the questionnaires were given to the participants to complete via self‐reporting. Approximately 25 individuals, including 9 from the cosmetic surgical group and 16 from the comparison group, withdrew from the study. Nevertheless, sampling continued until the calculated sample size was achieved.

### 2.3 Data Collection Instruments

In this study, data were collected using a structured questionnaire consisting of the following components:

1. Demographic Questionnaire: This section included questions regarding participants' age, education level, occupation, marital status, and type of cosmetic surgeries undergone.
2. Body Appreciation Scale (BAS): The Body Appreciation Scale is used to assess positive body image. Avalos and colleagues (2005) [23] developed and standardized this scale in the United States, where it demonstrated high internal consistency (Cronbach's *α* = 0.93). The item‐total correlations ranged from 0.46 or higher. The Body Appreciation Scale (BAS) consists of 13 items scored on a 5‐point Likert scale ranging from 1 (never) to 5 (always). The total BAS score was calculated by summing the responses to all 13 items, resulting in a possible score ranging from 13 to 65. Higher scores indicate greater body appreciation. Specifically, the scale measures how individuals value their bodies by evaluating the following dimensions: Preservation of favored body parts, Acceptance of one's body despite imperfections and non‐ideal shape or weight, Acceptance of one's body based on physical needs and engagement in behaviors promoting health, and Protection of one's body by rejecting unrealistic media images of thin, idealized models. These dimensions, which represent unconditional respect and appreciation for the body, are collectively referred to as body appreciation. In Iran, Hasheminejad and colleagues psychometrically evaluated this scale, reporting a Cronbach's *α* of 0.88, which indicates acceptable reliability within the Iranian population. Exploratory factor analysis revealed a two‐factor structure explaining 55.12% of the variance in body appreciation scores [24].
3. Body Image Concerns Inventory (BICI): The Body Image Concerns Inventory (BICI) was developed by Littleton and colleagues in 2005 [25]. It evaluates concerns related to physical appearance across multiple domains, including dissatisfaction and shame regarding one's appearance, checking and concealing perceived flaws, and the extent to which appearance‐related worries interfere with social functioning. The inventory consists of 19 items rated on a 5‐point Likert scale ranging from 1 (Never) to 5 (Always). In Iran, Tajikzadeh et al., (2013) reported an internal consistency reliability (Cronbach's *α*) of 0.95 [26]. The total score is obtained by summing the scores of all items, resulting in a possible range of 19 to 95. Interpretation of the total score is as follows: 19–37: Very low or no body image concerns 38–52: Low body image concerns 53–69: Moderate body image concerns 70 and above: High body image concerns The BICI has two subscales: Dissatisfaction and shame regarding appearance, checking and concealing perceived flaws (items 1, 3, 5, 8, 9, 14, 15, 16, 17, 18, 19), Interference of appearance‐related concerns in social functioning (items 2, 4, 6, 7, 10, 11, 12, 13) [25].
4. Short form health‐related quality‐of‐life questionnaire (SF‐12): To assess participants' health‐related quality of life. This instrument has been widely used in various studies [27]. Montazeri et al. (2009) [28] examined its validity and reliability in Iran, reporting Cronbach's *α* values of 0.73 for the physical component and 0.72 for the mental component. Physical Component Summary (PCS‐12) and Mental Component Summary (MCS‐12) scores were calculated according to the standard norm‐based scoring procedure described. Scoring followed the standard norm‐based algorithm developed by Ware et al. [27] in which item responses are converted to indicator variables and transformed to a mean of 50 and a standard deviation of 10. Higher scores indicate better health‐related quality of life. In addition to the two summary scores, an overall SF‐12 score ranging from 0 to 100 was computed for descriptive purposes, consistent with common reporting practices in the Iranian literature.
5. General Health Questionnaire (GHQ‐28): The General Health Questionnaire (GHQ‐28) is a 28‐item screening tool designed to detect common mental disorders. It uses a 4‐point Likert‐type response format scored from 0 to 3. Total scores range from 0 to 84, with higher scores indicating poorer mental and physical health status. In Iran, the validity and reliability of the GHQ‐28 have been previously established and confirmed [29].

### 2.4 Data Analysis Method

The research findings were statistically analyzed using IBM SPSS Statistics for Windows, Version 24.0 (IBM Corp., Armonk, NY, USA). For descriptive statistics, the mean and standard deviation were used for quantitative variables, while frequency and percentage were used for qualitative variables. Although the data showed slight skewness, they were considered approximately normal since both skewness and kurtosis values fell within the range of −1 to +1. To compare demographic characteristics and mean scores of the main study variables, including health‐related quality of life, general health, body appreciation, and body image concerns across the three groups, the *χ* ^2^ test, Fisher's exact test, and one‐way ANOVA were employed. The primary analyses were the one‐way ANOVA comparisons of the three study groups on the four main outcomes (SF‐12, GHQ‐28, BICI, and BAS). Post hoc Tukey's tests were used for pairwise comparisons between groups. Before conducting one‐way ANOVA, the assumption of homogeneity of variance was assessed using Levene's test. The assumption was met for all primary outcomes (*p* > 0.05).

Pearson's correlations and multiple linear regression models were considered secondary/exploratory analyses aimed at examining associations among the variables and adjusted associations with demographic factors. Multiple linear regression analysis was conducted to examine the adjusted associations between study group and the four main outcomes (health‐related quality of life [SF‐12], general health [GHQ‐28], body image concern [BICI], and body appreciation [BAS]) while controlling for demographic covariates. The study group was entered as two dummy variables, with the comparison group serving as the reference category (Operated vs Comparison and Candidate vs Comparison). Reference categories for the categorical covariates were university education, employed status, single marital status, and “enough” income. Before conducting multiple linear regression, the assumptions of linear regression were evaluated. Multicollinearity was assessed using variance inflation factors (VIFs), and no evidence of problematic multicollinearity was observed (all VIFs < 5). Normality and homoscedasticity of residuals were assessed using residual plots, and independence of errors was evaluated using the Durbin–Watson statistic. The assumptions were considered adequately satisfied. Variables included in the multiple regression models were selected based on their clinical relevance and evidence from previous studies. In all analyses, the significance level was set at *α* = 0.05, with a 95% confidence interval.

## 3 Results

A total of 375 women participated in this study. The mean age of participants was 29.41 years (range: 18–59). Significant differences were observed among the three groups regarding age, education, marital status, occupation, and household income. Most participants in the cosmetic surgery and surgery candidate groups were homemakers, whereas employment was more common in the Comparison group (Table 1).

**Table 1:** Comparison of demographic characteristics among women with previous cosmetic surgery, cosmetic surgery candidates, and the comparison group.

Variable	All *n* = 375	Operated *n* = 123	Cosmetic surgery candidates *n* = 138	Comparison group *n* = 114	*p*‐value
Women's Age (years) Mean (SD) *n* (%)	29.41 (7.69)	30.00 (9.92)	30.02 (5.72)	29.03 (6.87)	0.071^a^
< 20	14 (3.7)	7 (5.69)	3 (2.18)	4 (3.5)	
20–29	207 (55.2)	67 (54.48)	66 (47.82)	74 (64.91)	
30–39	114 (30.4)	25 (20.33)	61 (44.2)	28 (24.56)	
40–49	31 (8.3)	16 (13.00)	8 (5.80)	7 (6.15)	
> 50	9 (2.4)	8 (6.5)	0 (0)	1 (0.88)	
Women's Educational Status *n* (%)					
Secondary school	10 (2.7)	3 (2.44)	7 (5.08)	0 (0)	0.018^b^
Diploma	116 (30.9)	28 (22.76)	44 (31.88)	44 (38.60)	
University	249 (66.4)	92 (74.80)	87 63.04)	70 (61.40)	
Women's Employment Status *n* (%)					
Housewife	260 (69.3)	95 (77.23)	101 (73.18)	64 (56.14)	0.001^c^
Employed	115 (30.7)	28 (22.77)	37 (26.82)	50 (43.86)	
Marital status					0.001^c^
Single	151 (40.3)	53 (43.1)	69 (50)	29 (25.4)	
Married	224 (59.7)	70 (56.9)	69 (50)	85 (74.6)	
Income status *n* (%)					
More than enough	29 (7.74)	13 (10.6)	14 (10.2)	2 (1.8)	< 0.001^c^
enough	251 (66.93)	95 (77.2)	94 (68.1)	62 (54.4)	
Less than enough	95 (25.33)	15 (12.2)	30 (21.7)	50 (43.8)	
Kind of cosmetic surgery *n* (%)					
Nose	226 (60.5)	103 (83.7)	123 (89.2)	—	< 0.001
Face lift	15 (4.0)	14 (11.4)	1 (0.7)	—	
Blepharoplasty	20 (5.5)	6 (4.9)	14 (10.1)	—	

^a^ ANOVA.

^b^ Fisher's exact test.

^c^ *χ* ^2^.

Initial analysis of surgical characteristics revealed that approximately 60.5% of the participants had undergone rhinoplasty. Specifically, 83.7% and 89.1% of participants in the surgically treated group and the surgery candidate group, respectively, preferred rhinoplasty. Blepharoplasty was the second most frequently requested procedure (see Table 1). The mean time elapsed since surgery in the surgically treated group was 3.05 years (SD = 0.97).

### 3.1 Comparison of Total Scores on Health‐Related Quality of Life, General Health, Body Appreciation, and Body Image Concerns Among the Three Groups

According to Table 2, the mean total scores for health‐related quality of life, general health, body appreciation, and body image concerns were 56.18, 27.58, 50.25, and 41.11, respectively. One‐way ANOVA showed statistically significant differences among the three groups for total quality of life (*F* = 6.759, *p* < 0.001), GHQ‐28 (*F* = 18.166, *p* < 0.001), Body Image Concern Inventory (*F* = 5.602, *p* = 0.004), and Body Appreciation Scale (*F* = 3.242, *p* = 0.040).

**Table 2:** Comparison of the total main variable scores in the three groups.

Variable	All *n* = 375		Operated *n* = 123		Those who are undergoing surgery *n* = 138		Comparison group *n* = 114		Analysis of variance	
	Mean	SD	Mean	SD	Mean	SD	Mean	SD	*p*‐Value	*F*
Total Health‐related quality of life score	56.18	9.40	58.69	10.03	54.81	7.86	55.14	9.93	< 0.001	6.759
Physical component score	37.47	12.42	42.09	10.48	34.58	12.42	36.00	13.38	< 0.001	13.927
Mental component score	49.92	7.09	49.45	8.48	50.51	6.11	49.70	6.55	0.453	0.793
GHQ‐28 score	27.58	12.18	23.51	10.70	27.06	12.78	32.60	11.23	< 0.001	18.166
somatic symptoms	8.37	3.11	7.43	2.88	8.34	3.64	9.42	2.20	< 0.001	13.006
anxiety	7.32	4.53	5.54	3.83	8.47	5.23	7.85	3.68	< 0.001	15.909
social dysfunction	7.94	3.12	7.62	2.96	6.60	2.78	9.92	2.69	< 0.001	44.469
depression	3.92	4.49	2.90	4.13	3.63	3.23	5.38	5.69	< 0.001	9.945
Body image concern inventory	41.11	12.55	38.06	10.98	42.86	13.67	42.27	12.23	0.004	5.602
dissatisfaction with appearance	21.91	7.20	19.95	6.49	22.42	7.86	23.42	6.67	< 0.001	7.618
Concern about appearance	19.18	6.07	18.09	5.32	20.44	6.60	18.85	5.94	0.006	5.206
Body Appreciation Scale	50.25	8.78	50.91	7.17	48.25	9.05	51.35	9.81	0.040	3.242

Tukey's post hoc tests indicated that the operated group had significantly higher quality‐of‐life scores than both the candidate group (*p* < 0.05) and the comparison group (*p* < 0.05); no significant difference existed between candidates and the comparison group. For GHQ‐28, the operated group scored significantly lower (better psychological health) than both the candidate and comparison groups (both *p* < 0.05), while candidates and the comparison group did not differ significantly. For the Body Image Concern Inventory, the operated group showed significantly lower scores than the candidate group (*p* < 0.05); other pairwise comparisons were non‐significant. For the Body Appreciation Scale, the only significant pairwise difference was between the candidate group and the comparison group (*p* < 0.05) (Table 2).

### 3.2 Relationship Between the Main Study Variables and Predictive Factors

As shown in Table 3, Pearson's correlation analysis revealed statistically significant and inverse relationships between mean health‐related quality of life scores and both general health scores (*r* = −0.426, *p* < 0.001, medium effect) and body image concerns scores (*r* = −0.269, *p* < 0.001, small effect). In addition, mean quality of life scores and body appreciation scores were significantly positively correlated (*r* = 0.434, *p* < 0.001, medium effect). Furthermore, a significant negative correlation was found between general health and body appreciation (*r* = −0.446, *p* < 0.001, medium effect). In contrast, a significant positive correlation was found between general health and body image concerns (*r* = 0.465, *p* < 0.001, medium effect). The results also indicated a strong and significant negative correlation between body image concerns and body appreciation (*r* = −0.625, *p* < 0.001, strong effect).

**Table 3:** Correlations between the main variables of the study.

Variable	1	2	3	4
1‐Total Health‐related quality of life score (SF12)	1	−0.426*	−0.269*	0.434*
2‐Total General Health score (GH)		1	0.465*	−0.446*
3‐Body Image Concern Inventory (BICI)			1	−0.625*
4‐Body Appreciation Scale (BAS)				1

* *p* < 0.001.

Finally, multiple linear regression was conducted to examine how demographic variables were associated health‐related quality of life. In Model 1, six independent variables, including group membership (surgically treated, surgery candidate, and Comparison group), age, educational status, occupation, marital status, and income level, explained only 8.1% of the variance in health‐related quality of life (*R* ^2^adj = 0.081, *p* < 0.001). In the multiple linear regression model predicting quality of life (SF‐12), after dummy‐coding the group variable (comparison group as reference), women with a history of cosmetic surgery had significantly higher quality‐of‐life scores than the comparison group (*B* = 2.599, *β* = 0.130, *p* = 0.044). Surgery candidates did not differ significantly from the comparison group (*p* = 0.156). Being a housewife was associated with lower quality of life (*β* = −0.155, *p* = 0.007), while being married was associated with higher quality of life (*β* = 0.150, *p* = 0.009). Participants reporting “more than enough” income had significantly lower quality‐of‐life scores compared with those reporting “enough” income (*β* = −0.208, *p* = 0.026). Educational level was not a significant predictor in this model.

In Model 2, which examined the same six variables as predictors of general health, the model explained 12.1% of the variance in general health scores (*R* ^2^adj = 0.121, *p* < 0.001). In the multiple linear regression model predicting general health (GHQ‐28), dummy‐coded group membership was a significant predictor. Women with a history of cosmetic surgery had substantially lower GHQ‐28 scores (better psychological health) than the comparison group (*B* = −8.560, *β* = −0.330, *p* < 0.001). Surgery candidates also had significantly lower scores than the comparison group (*B* = −5.078, *β* = −0.201, *p *= 0.002). None of the demographic variables (education, employment, marital status, age, or income level) reached statistical significance in this model (all *p* > 0.05), although participants reporting “less than enough” income showed a trend toward higher (poorer) GHQ‐28 scores.

Model 3 indicated that the six independent variables predicted 14.6% of the variance in body image concerns (*R* ^2^adj = 0.146, *p* < 0.001). In the multiple linear regression model predicting body image concern (BICI), group membership was not a significant predictor after dummy coding (Operated vs. Comparison: *p* = 0.171; Candidate vs. Comparison: *p* = 0.368). Lower educational level was associated with greater body‐image concern: secondary‐school education (*β* = 0.189, *p* < 0.001) and diploma education (*β* = 0.119, *p* = 0.028) were both associated with higher BICI scores relative to university education. Being a housewife was associated with lower body‐image concern– −0.180, *p* = 0.001). Participants reporting “less than enough” income had substantially higher BICI scores (*β* = 0.351, *p* < 0.001). Age and marital status were not significant predictors.

Finally, in Model 4, in the multiple linear regression model predicting body appreciation (BAS), surgery candidates had significantly lower body‐appreciation scores than the comparison group (*B* = −3.162, *β* = −0.174, *p* = 0.007). The operated group did not differ significantly from the comparison group (*p* = 0.346). Both “less than enough” income (*β* = −0.434, *p* < 0.001) and “more than enough” income (*β* = −0.477, *p* < 0.001) were associated with substantially lower body‐appreciation scores relative to “enough” income. Educational level, employment status, marital status, and age were not significant predictors in this model (Table 4).

**Table 4:** Multiple linear regression analysis of the main variables.

Associated factors	*R*	*R* ^2^	*R* ^2^_adj_	*F*	*B*	SE	*β*	*t*	*p*	95% CI for *B*
**Model 1: SF12**										
	0.321	0.103	0.081	4.622					< 0.001	
Constant					58.615	2.174	—	26.964	< 0.001	54.341 to 62.890
Operated (vs. comparison)					2.599	1.283	0.130	2.025	0.044	0.075 to 5.123
Candidate (vs. Comparison)					−1.769	1.245	−0.091	−1.421	0.156	−4.218 to 0.679
Education—Secondary school					5.581	3.137	0.096	1.779	0.076	−0.588 to 11.751
Education—Diploma					1.570	1.140	0.077	1.378	0.169	−0.671 to 3.811
Housewife (vs. Employed)					−3.156	1.163	−0.155	−2.715	0.007	−5.443 to −0.870
Married (vs. Single)					2.863	1.086	0.150	2.635	0.009	0.727 to 4.999
Income—Less than enough					−2.664	2.107	−0.123	−1.265	0.207	−6.807 to 1.479
Income—More than enough					−4.147	1.854	−0.208	−2.237	0.026	−7.792 to −0.502
**Model 2: General health**										
	0.347	0.121	0.099	5.559					< 0.001	
Constant					28.404	3.685	—	7.709	< 0.001	21.158 to 35.650
Operated (vs. Comparison)					−8.560	1.639	−0.330	−5.222	< 0.001	−11.784 to −5.337
Candidate (vs. Comparison)					−5.078	1.590	−0.201	−3.194	0.002	−8.205 to −1.951
Education—Secondary school					−1.387	4.009	−0.018	−0.346	0.730	−9.271 to 6.498
Education—Diploma					−2.556	1.457	−0.097	−1.754	0.080	−5.422 to 0.310
Housewife (vs. Employed)					−1.252	1.485	−0.047	−0.843	0.400	−4.173 to 1.669
Married (vs. Single)					1.031	1.596	0.042	0.646	0.519	−2.108 to 4.170
Income—Less than enough					7.233	2.690	0.258	2.688	0.008	1.942 to 12.524
Income—More than enough					4.142	2.367	0.160	1.750	0.081	−0.513 to 8.797
Age (years)					0.001	0.092	0.001	0.016	0.987	−0.180 to 0.183
**Model 3: BICI**										
	0.416	0.173	0.153	8.486					< 0.001	
Constant					39.393	3.682	—	10.699	< 0.001	32.152 to 46.633
Operated (vs Comparison)					−2.247	1.638	−0.084	−1.372	0.171	−5.468 to 0.974
Candidate (vs Comparison)					1.433	1.589	0.055	0.902	0.368	−1.691 to 4.558
Education—Secondary school					14.726	4.006	0.189	3.676	< 0.001	6.847 to 22.604
Education—Diploma					3.217	1.456	0.119	2.209	0.028	0.353 to 6.081
Housewife (vs Employed)					−4.895	1.484	−0.180	−3.298	0.001	−7.813 to −1.976
Married (vs Single)					−0.898	1.595	−0.035	−0.563	0.574	−4.035 to 2.238
Income—Less than enough					10.121	2.688	0.351	3.765	< 0.001	4.835 to 15.408
Income—More than enough					2.854	2.365	0.107	1.207	0.228	−1.797 to 7.505
Age (years)					−0.071	0.092	−0.043	−0.765	0.445	−0.252 to 0.111
**Model 4: BAS**										
	0.291	0.085	0.062	3.751					< 0.001	
Constant					61.268	2.711	—	22.601	< 0.001	55.938 to 66.599
Operated (vs. Comparison)					−1.138	1.206	−0.061	−0.943	0.346	−3.509 to 1.234
Candidate (vs. Comparison)					−3.162	1.170	−0.174	−2.703	0.007	−5.463 to −0.862
Education—Secondary school					1.296	2.950	0.024	0.439	0.661	−4.504 to 7.097
Education—Diploma					−0.464	1.072	−0.024	−0.433	0.666	−2.572 to 1.644
Housewife (vs Employed)					−0.344	1.093	−0.018	−0.315	0.753	−2.493 to 1.805
Married (vs Single)					−1.008	1.174	−0.056	−0.858	0.391	−3.317 to 1.302
Income—Less than enough					−8.766	1.979	−0.434	−4.429	< 0.001	−12.658 to −4.873
Income—More than enough					−8.894	1.741	−0.477	−5.107	< 0.001	−12.319 to −5.470
Age (years)					−0.023	0.068	−0.020	−0.341	0.733	−0.157 to 0.110

## 4 Discussion

The present study aimed to evaluate and compare the quality of life, general health, body appreciation, and body image concerns among three groups: women with a history of cosmetic surgery, women who were candidates for cosmetic surgery, and members of the Comparison group. The findings revealed statistically significant differences between the groups in terms of scores on these variables, with surgically treated individuals achieving relatively better scores across all domains.

The regression models explained a small to modest proportion of the variance (adjusted *R* ^2^ ranging from 0.011 to 0.146), suggesting that additional unmeasured factors may contribute to the study outcome. This finding suggests that quality of life and psychological well‐being are multifactorial constructs influenced by additional psychological, social, and environmental factors that were not assessed in this study. Therefore, these results should be interpreted with caution.

To achieve the study objectives, an initial assessment of participants' health‐related quality of life showed moderate and unsatisfactory scores overall. A comparison across the three groups revealed a statistically significant difference, with surgically treated individuals scoring higher on health‐related quality of life than the other two groups, while the lowest scores were observed among cosmetic surgical candidates. Regarding specific domains of health‐related quality of life, a statistically significant difference was found in the physical health domain among the three groups, with cosmetic surgical candidates receiving the lowest scores. Although cosmetic surgical candidates also scored lowest in the mental health domain, no statistically significant difference was observed in this area. According to our findings, women with a history of cosmetic surgery reported higher quality‐of‐life scores than cosmetic surgical candidates and women from the Comparison group. However, because this study employed a cross‐sectional design, these findings indicate an association rather than demonstrating that cosmetic surgery caused improvements in quality of life. Studies conducted in Iran on women and the Comparison group have similarly reported moderate levels of quality of life [30, 31], which may be influenced by individual factors such as education level, socioeconomic status, self‐esteem, and satisfaction with appearance [32]. These findings are consistent with recent systematic reviews, which report improvements in body image, self‐esteem, and procedure‐specific satisfaction after cosmetic surgery, while emphasizing that evidence for sustained improvements in overall psychological health and quality of life remains inconclusive because of the limited quality and heterogeneity of existing studies [15, 33, 34]. Findings from various international studies also support improvements in quality of life after facial cosmetic surgery [35, 36, 37]. One German study that assessed quality of life 3 to 13 years after rhinoplasty reported long‐term improvement [33]. However, some studies have shown no significant change and even a decline in quality of life after rhinoplasty [7, 20]. In those cases, the decline was attributed to unnecessary surgery and medical errors. Additionally, impaired function of the upper airways is a known factor contributing to the negative effects of rhinoplasty on quality of life. In one such study, although quality of life initially decreased after rhinoplasty, it gradually improved over time [38]. Considering the cultural context in Iran, where women are required to wear the hijab, the face becomes the primary visible aspect of appearance. As a result, facial cosmetic surgery are more popular and tend to lead to improvements in post‐surgery quality of life.

Psychological and Psychosocial Evaluation The next stage of the analysis focused on the psychological and psychosocial well‐being of the female participants. GHQ‐28 scores differed significantly across the three groups. The Comparison group and surgery candidate groups had higher GHQ‐28 scores, indicating poorer psychological health than women with a history of cosmetic surgery. Specifically, both the Comparison group and the cosmetic surgical candidate group scored higher in terms of psychological distress compared to the surgically treated group. These higher scores reflected poorer mental health outcomes related to anxiety, social functioning, depression, and somatic symptoms. Except for anxiety, the Comparison group showed worse scores across most domains. Moreover, psychological health scores were found to be associated with participants' income status. These findings align with those of other studies [24, 39]. Previous longitudinal and prospective studies have reported improvements in several psychological outcomes following cosmetic surgery [24]. In the present cross‐sectional study, women with a history of cosmetic surgery exhibited more favorable psychological health scores than the comparison groups; however, these findings should not be interpreted as evidence that cosmetic surgery caused these differences. A systematic review evaluating 16 published articles reported that cosmetic surgery has a positive impact on various psychological factors, such as depression and anxiety. Evidence indicates notable improvements in mental health and social functioning, along with a reduction in psychosocial distress [40]. This improvement in psychological well‐being may also influence quality of life, as a positive correlation between these two variables was observed in our study.

Our findings regarding body image and fear of physical appearance revealed that the surgically treated group had significantly lower mean total BICI (Body Image Concerns Inventory) scores compared to the other two groups. Statistically significant differences were observed, particularly in the subscale measuring dissatisfaction with physical appearance. The Comparison group and cosmetic surgical candidates expressed greater dissatisfaction than the surgically treated group. Notably, concerns about physical appearance were greatest among cosmetic surgical candidates.

Body‐appreciation scores were lowest among cosmetic surgical candidates; the operated and comparison groups showed comparable levels. According to previous studies, dissatisfaction with physical appearance is a major motivator for seeking cosmetic surgery [33, 41, 42]. Researchers have indicated that cosmetic surgeries are primarily performed to increase satisfaction and boost self‐esteem. It is believed that individuals seeking cosmetic surgery are psychologically healthy, and their primary motivation is to improve their appearance [41].

Sarwer and Whitaker reported improvements in body image following cosmetic surgery in longitudinal research [43]. Consistent with these findings, women with a history of cosmetic surgery in our study demonstrated more favorable body image scores than the comparison groups. However, because of the cross‐sectional design, our results demonstrate an association rather than a causal effect. Body appreciation has been linked to positive self‐concept, life satisfaction, and reduced levels of anxiety and depression. According to cognitive‐behavioral models, how individuals think about their bodies is closely connected to psychological difficulties, a relationship also observed in our study.

A possible explanation for these findings is that dissatisfaction with body image may be influenced by cultural norms and peer groups, leading to distorted perceptions, negative thoughts, and emotions, which can ultimately contribute to psychosocial problems. Our findings should also be interpreted within the broader sociocultural context. Recent regional evidence suggests that increasing sociocultural pressures, social media exposure, and evolving beauty ideals may substantially influence body image perceptions and motivations for cosmetic surgery. These findings further support the importance of comprehensive preoperative psychological assessment to identify unrealistic expectations and psychological vulnerabilities before cosmetic surgery [5, 33, 44].

The relatively poorer psychological outcomes observed in the comparison group should also be interpreted with caution. Although these women had not sought cosmetic surgery, psychological well‐being and body image concerns may be influenced by unmeasured factors such as sociocultural pressures, appearance ideals, socioeconomic characteristics, and individual psychological traits. In addition, residual confounding cannot be excluded because these variables were not assessed. Therefore, these findings should be considered associative rather than indicative of a specific underlying cause.

The interpretation of our findings should also consider the possibility of selection bias. Women who had previously undergone cosmetic surgery, women seeking surgery, and women from the Comparison group may represent distinct subgroups with different socioeconomic backgrounds, expectations, and psychological profiles. In our sample, significant differences were observed across groups in income, education, employment, and marital status, all of which may influence quality of life and mental health independently of cosmetic surgery. Moreover, the operated group had undergone surgery several years before the study, whereas the candidate group had not yet undergone surgery. Therefore, some of the observed differences may reflect pre‐existing characteristics or long‐term adaptation rather than the direct effect of cosmetic surgery itself.

### 4.1 Strengths, Limitations, and Future Research Directions

Unlike previous studies that were often designed as pre‐ and post‐intervention designs or compared only two groups, surgically treated individuals and surgical candidates, the present study aimed to compare quality of life and psychological factors across three groups, including a comparison group. This comprehensive approach represents one of the key strengths of our study.

However, due to certain limitations, particularly the use of convenience sampling, which may limit the generalizability of the findings to other regions and healthcare settings may be limited. The three study groups also differed in their stage of exposure to cosmetic surgery: women in the operated group had undergone surgery an average of approximately 3 years earlier, surgery candidates had not yet undergone the procedure, and the comparison group was recruited through a different pathway. These groups may therefore have differed in socioeconomic status, expectations, motivations, body image concerns, and other psychological characteristics independently of surgery. Although some demographic variables were included in the regression analyses, residual confounding from unmeasured factors cannot be excluded. Consequently, the observed differences in quality of life and psychological outcomes should be interpreted with caution. Future studies should employ multicenter recruitment and probability‐based sampling methods. An important limitation of this study is its cross‐sectional design, which precludes causal inference. Although significant differences were observed between the study groups, it cannot be determined whether cosmetic surgery itself contributed to these differences or whether participants differed in psychological characteristics and quality of life before surgery. The operated group differed from the candidate and comparison groups not only in surgical exposure but also potentially in socioeconomic status, marital status, employment, expectations, motivation, body‐image concerns, and other unmeasured characteristics. In addition, the operated group had undergone surgery a mean of approximately 3 years earlier. Therefore, selection effects, pre‐existing differences, and long‐term adaptation cannot be disentangled from any potential effect of surgery itself. Prospective longitudinal studies that assess participants before and after cosmetic surgery are needed to clarify the temporal relationship between cosmetic surgery and psychological outcomes. In addition, several potentially important confounding variables, including BMI, body dysmorphic disorder, postoperative complications, satisfaction with surgical outcomes, and previous cosmetic surgery, were not assessed and may have influenced the observed associations. Future studies should incorporate these variables to better understand the factors associated with psychological outcomes after cosmetic surgery. Furthermore, qualitative studies exploring the deep‐rooted motivations behind cosmetic surgery could provide richer insights into participants' experiences and decision‐making processes.

## 5 Conclusion

Women with a history of cosmetic surgery reported more favorable health‐related quality of life, general health, and body‐image‐concern scores than surgery candidates and the comparison group. Body‐appreciation scores were lowest among surgery candidates, with no significant difference between the operated and comparison groups. The regression models revealed only modest associations and explained a limited proportion of the variance in the outcomes. However, because this study was cross‐sectional, these findings should be interpreted as associations rather than evidence that cosmetic surgery caused these differences. Given the observed differences in psychological well‐being and health‐related quality of life among the participant groups, careful attention to the psychological status of women considering cosmetic surgery may be warranted. Routine psychological assessment and evaluation of patients' motivations before surgery could help clinicians identify individuals with unrealistic expectations or significant body‐image concerns. However, the present study does not provide evidence that such assessment improves surgical outcomes or prevents inappropriate procedures; these potential benefits require evaluation in future prospective research.

## Author Contributions

**Azita Fathnezhad‐Kazemi:** conceptualization, investigation, methodology, validation, visualization, software, formal analysis, project administration. **Asal Rahbar Zeraati:** data curation, conceptualization, investigation.

## Funding

The authors have nothing to report.

## Ethics Statement

Written informed consent was obtained from each participant before the completion of the survey. This study was approved by the Ethics Committee of the Islamic Azad University of Tabriz Medical Sciences, Iran (code number: IR.IAU.TABRIZ.REC.1402.075). All the methods were carried out according to relevant guidelines and regulations.

## Consent

The authors have nothing to report.

## Conflicts of Interest

The authors declare no conflicts of interest.

## Declaration of Generative AI and AI‐Assisted Technologies in the Writing Process

The authors declared that OpenAI was used to refine the manuscript's English language.

## Transparency Statement

Azita Fathnezhad‐Kazemi affirms that this manuscript is an honest, accurate, and transparent account of the study being reported; that no important aspects of the study have been omitted; and that any discrepancies from the study as planned (and, if relevant, registered) have been explained.

## Data Availability

The data that support the findings of this study are available from the corresponding author upon reasonable request.

## References

[1] H. A. Al Ghadeer , M. A. AlAlwan , M. A. AlAmer , et al., “Impact of Self‐Esteem and Self‐Perceived Body Image on the Acceptance of Cosmetic Surgery,” Cureus 13, no. 10 (2021): e18825. 34804682 10.7759/cureus.18825 PMC8592260

[2] A. Sheikhi , B. Saadatifar , H. Mastaelizadeh , Z. Zahedi , M. Sari , and H. Sheikhi , “Comparison of Body Image and General Health of Subjects Undergoing Surgery and Applying for Cosmetic Nose Surgery With Normal People in Kerman City in 2016,” Supplement, Journal of Advanced Pharmacy Education & Research 9, no. S2 (April/June 2019): 129–134.

[3] M. Abbasi , A. Aghighi , P. Porzoor , and M. Dehqan , “Comparison of Early Maladaptive Schemas and Psychological Well‐Being in Women Undergoing Cosmetic Surgery and Normal Women,” Journal of Research and Health 7, no. 3 (2017): 841–849.

[4] Y. Zhang , M. Jiang , J. Liu , and B. Liu , “Pursuing Beauty: Socio‐Cultural and Labor‐Economic Determinants of Cosmetic Surgery Consideration Among Female College Students in China,” BMC Psychology 12, no. 1 (2024): 519. 39350303 10.1186/s40359-024-02016-w PMC11440754

[5] M. Asadi , M. Salehi , M. Sadooghi , and A. A. Ebrahimi , “Self‐Esteem and Attitude Toward Body Appearance Before and After Cosmetic Rhinoplasty,” Iranian Journal of Psychiatry & Clinical Psychology 19, no. 1 (2013): 20–33.

[6] H. Najjaran Toussi and H. Shareh , “Changes in the Indices of Body Image Concern, Sexual Self‐Esteem and Sexual Body Image in Females Undergoing Cosmetic Rhinoplasty: A Single‐Group Trial,” Aesthetic Plastic Surgery 43 (2019): 771–779. 30805690 10.1007/s00266-019-01336-2

[7] R. Zojaji , E. Sobhani , M. Meshkat , and M. Javanbakht , “Quality of Life After Cosmetic Rhinoplasty in Iran: A Systematic Review,” Journal of Fundamentals of Mental Health 20, no. 5 (2018): 265–269.

[8] L. Esmalian Khamseh and M. Nodargahfard , “The Effect of Cosmetic Surgery on Sexual Self‐Esteem: Attitudes Toward Body Image and Well‐Being in Married Women,” World Journal of Plastic Surgery 9, no. 2 (2020): 153–159. 32934926 10.29252/wjps.9.2.153 PMC7482527

[9] A. Salahian , “Comparison of Mental Health and Self‐Image Between the Applicants and Non‐Applicants of Cosmetic Surgery,” Journal of Research and Health 9, no. 7 (2019): 686–691.

[10] J. F. Sobanko , J. Dai , J. M. Gelfand , D. B. Sarwer , and I. Percec , “Prospective Cohort Study Investigating Changes in Body Image, Quality of Life, and Self‐Esteem Following Minimally Invasive Cosmetic Procedures,” Dermatologic Surgery 44, no. 8 (2018): 1121–1128. 29659404 10.1097/DSS.0000000000001523

[11] V. A. Picavet , E. P. Prokopakis , L. Gabriëls , M. Jorissen , and P. W. Hellings , “High Prevalence of Body Dysmorphic Disorder Symptoms in Patients Seeking Rhinoplasty,” Plastic and Reconstructive Surgery 128, no. 2 (2011): 509–517. 21788842 10.1097/PRS.0b013e31821b631f

[12] L. Woertman and F. Van den Brink , “Body Image and Female Sexual Functioning and Behavior: A Review,” Journal of Sex Research 49, no. 2–3 (2012): 184–211. 22380588 10.1080/00224499.2012.658586

[13] N. Yazdani , S. V. Hosseini , M. Amini , Z. Sobhani , F. Sharif , and H. Khazraei , “Relationship Between Body Image and Psychological Well‐Being in Patients With Morbid Obesity,” International Journal of Community Based Nursing and Midwifery 6, no. 2 (2018): 175–184. 29607346 PMC5845121

[14] Y. Khodabandeloo , J. Fat'h‐Abadi , N. Motamed‐Yeganeh , and S. Yadollahi , “Factor Structure and Psychometric Properties of the Multidimensional Body‐Self Relations Questionnaire (MBSRQ) in Female Iranian University Students,” Practice in Clinical Psychology 7, no. 3 (2019): 187–196.

[15] K. M. Garbett , N. Paraskeva , P. White , et al., “The Psychosocial Outcomes Following Cosmetic Surgery Are Largely Unknown: A Systematic Review,” Journal of Plastic, Reconstructive & Aesthetic Surgery: JPRAS 104 (2025): 282–297. 40156948 10.1016/j.bjps.2025.03.013

[16] Z. Khanjani , J. Babapour , and G. Saba , “Investigating Mental Status and Body Image in Cosmetic Surgery Applicants in Comparison With Non‐Applicants,” SSU_Journals 20, no. 2 (2012): 237–248.

[17] S. A. Cook , R. Rosser , and P. Salmon , “Is Cosmetic Surgery an Effective Psychotherapeutic Intervention? A Systematic Review of the Evidence,” Journal of Plastic, Reconstructive & Aesthetic Surgery: JPRAS 59, no. 11 (2006): 1133–1151. 17046622 10.1016/j.bjps.2006.03.047

[18] M. J. Fatemi , F. Rajabi , S. J. Moosavi , and M. Soltani , “Quality of Life Among Iranian Adults Before and After Rhinoplasty,” Aesthetic Plastic Surgery 36 (2012): 448–452. 21993575 10.1007/s00266-011-9820-y

[19] N. A. Papadopulos , L. Kovacs , S. Krammer , P. Herschbach , G. Henrich , and E. Biemer , “Quality of Life Following Aesthetic Plastic Surgery: A Prospective Study,” Journal of Plastic, Reconstructive & Aesthetic Surgery: JPRAS 60, no. 8 (2007): 915–921. 17379593 10.1016/j.bjps.2007.01.071

[20] R. Zojaji , M. Keshavarzmanesh , H. Arshadi , M. Farsi Baf , and S. Esmaeilzadeh , “Quality of Life in Patients Who Underwent Rhinoplasty,” Facial Plastic Surgery 30, no. 05 (2014): 593–596. 25397717 10.1055/s-0034-1393699

[21] M. Ahmadpanah , S. Mosavi , M. Dallband , M. Zandi , M. Saleh , and M. Nazaribadie , “The Study of Psychological Characteristics, Body Image, Quality of Life, and General Health of Rhinoplasty Applicants,” Avicenna Journal of Neuro Psycho Physiology 4, no. 3 (2017): 103–112.

[22] M. Rashidifar and M. Karami , “Early Maladaptive Schemas, Body Dysmorphic Disorder (BDD) and the Mediation Role of Rumination and Emotional Cognitive Regulation: A Focus on Cosmetics Surgery Applicants,” Proceedings of the International Conference on Research in Psychology 2 (2025): 29–43.

[23] L. Avalos , T. L. Tylka , and N. Wood‐Barcalow , “The Body Appreciation Scale: Development and Psychometric Evaluation,” Body Image 2, no. 3 (2005): 285–297. 18089195 10.1016/j.bodyim.2005.06.002

[24] N. F. S. Hashemi , S. Mantashloo , D. S. Izadi , and R. Roshan , “Determine the Psychometric Properties of the Body Appreciation Scales in Iranian Samples,” 4, no. 3 (2016): 65–71.

[25] H. L. Littleton , D. Axsom , and C. L. S. Pury , “Development of the Body Image Concern Inventory,” Behaviour Research and Therapy 43, no. 2 (2005): 229–241. 15629752 10.1016/j.brat.2003.12.006

[26] F. Tajikzadeh , R. Sadeghi , and F. Raiskariman , “The Relationship Between Body Dysmorphic Concern and Rumination Among the Girl University Students,” Sadra Medical Journal 1, no. 3 (2013): 189–198.

[27] J. E. Ware , M. Kosinski , and S. D. Keller , “A 12‐Item Short‐Form Health Survey: Construction of Scales and Preliminary Tests of Reliability and Validity,” Medical Care 34, no. 3 (1996): 220–233. 8628042 10.1097/00005650-199603000-00003

[28] A. Montazeri , M. Vahdaninia , S. J. Mousavi , and S. Omidvari , “The Iranian Version of 12‐Item Short Form Health Survey (SF‐12): Factor Structure, Internal Consistency and Construct Validity,” BMC Public Health 9 (2009): 341. 19758427 10.1186/1471-2458-9-341 PMC2749829

[29] S. Taghavi , “Validity and Reliability of the General Health Questionnaire (GHQ‐28) in College Students of Shiraz University,” Journal of Psychology 5, no. 4 (2002): 381–398.

[30] A. Beresniak , J. P. Auray , G. Duru , et al., “Quality of Life Assessment in Cosmetics: Specificity and Interest of the International BeautyQol Instrument,” Journal of Cosmetic Dermatology 14, no. 3 (2015): 260–265. 26133392 10.1111/jocd.12156

[31] A. H. Mohammed , B. A. R. Hassan , A. M. Wayyes , et al., “Exploring the Quality of Life of Cosmetic Users: A Cross‐Sectional Analysis From Eight Arab Countries in the Middle East,” Journal of Cosmetic Dermatology 22, no. 1 (2023): 296–305. 35567513 10.1111/jocd.15085 PMC10286760

[32] M. K. Jamal , F. R. Moghadam , B. H. Muhammadamin Malkari , et al., “Factors Influencing the Quality of Life of Postpartum Women: A Systematic Review,” Current Psychology 44, no. 8 (2025): 7326–7349.

[33] L. A. Borujeni , S. Pourmotabed , Z. Abdoli , et al., “A Comparative Analysis of Patients' Quality of Life, Body Image and Self‐Confidence before and After Aesthetic Rhinoplasty Surgery,” Aesthetic Plastic Surgery 44 (2020): 483–490. 31832733 10.1007/s00266-019-01559-3

[34] R. Dreher , C. Blaya , J. L. C. Tenório , R. Saltz , P. B. Ely , and Y. A. Ferrão , “Quality of Life and Aesthetic Plastic Surgery: A Systematic Review and Meta‐Analysis,” Plastic and Reconstructive Surgery—Global Open 4, no. 9 (2016): e862. 27757327 10.1097/GOX.0000000000000833 PMC5054993

[35] N. A. Papadopulos , J. Liebmann , M. Kloeppel , et al., “Quality of Life After Rhinoplasty: A Prospective Study,” Facial Plastic Surgery 37, no. 05 (2021): 639–645. 33706388 10.1055/s-0041-1725174

[36] R. Xavier , S. Azeredo‐Lopes , D.‐J. Menger , H. C. de Carvalho , and J. Spratley , “Generic Health‐Related Quality‐Of‐Life Changes After Rhinoplasty: A Prospective Study With Long‐Term Results,” Facial Plastic Surgery 39, no. 02 (2023): 164–172. 36037858 10.1055/a-1932-8563

[37] K. P. Luong , H. P. Slijper , B. Stubenitsky , S. Hummelink , and D. Ulrich , “Changes in Patient‐Reported Satisfaction and Quality‐Of‐Life 6 Months After Rhinoplasty,” Journal of Plastic, Reconstructive & Aesthetic Surgery: JPRAS 91 (2024): 325–334. 38442513 10.1016/j.bjps.2024.02.038

[38] M. Mohammadshahi , A. Pourreza , P. H. Orojlo , M. Mahmoodi , and F. Akbari , “Rhinoplasty as a Medicalized Phenomenon: A 25‐center Survey on Quality of Life Before and After Cosmetic Rhinoplasty,” Aesthetic Plastic Surgery 38 (2014): 615–619. 24902906 10.1007/s00266-014-0323-5

[39] K. Kosidou , C. Dalman , M. Lundberg , J. Hallqvist , G. Isacsson , and C. Magnusson , “Socioeconomic Status and Risk of Psychological Distress and Depression in the Stockholm Public Health Cohort: A Population‐Based Study,” Journal of Affective Disorders 134, no. 1–3 (2011): 160–167. 21665286 10.1016/j.jad.2011.05.024

[40] O. Katamanin , S. Saini , and M. Jafferany , “Psychological Implications and Quality of Life After Cosmetic Rhinoplasty: A Systematic Review,” Discover Psychology 4, no. 1 (2024): 16.

[41] A. Masoudzadeh , M. Karkhaneh Yousefi , and A. Tirgiri , “A Comparison of Personality Pattern and General Health Condition Between Individuals Seeking Cosmetic Nose Surgery and Those of the Control Group,” Daneshvar Medicine 16, no. 3 (2020): 53–58.

[42] A. Rahman , “Cultural Perspectives on Rhinoplasty: Trends Around the World,” Online Journal of Otolaryngology 14, no. 6 (2024): 417.

[43] D. Sarwer , L. Whitaker , T. Wadden , and M. Pertschuk , “Body Image Dissatisfaction in Women Seeking Rhytidectomy or Blepharoplasty,” Aesthetic Surgery Journal 17, no. 4 (1997): 230–234. 19327720 10.1016/s1090-820x(97)80004-0

[44] M. A. Patni , R. A. Salama , S. M. Vakhariya , et al., “Cosmetic Surgery in the Gulf Cooperation Council: Societal Shifts, Psychological Implications, and Policy Challenges: A Narrative Review,” Iranian Journal of Medical Sciences 51, no. 4 (2026): 224–235. 42100238 10.30476/ijms.2025.105988.4009 PMC13144777

